# Yellow and black-stained fingers in a patient with headache

**DOI:** 10.11604/pamj.2017.28.318.14333

**Published:** 2017-12-27

**Authors:** Fred Bernardes Filho, Frederico Macedo da Rocha

**Affiliations:** 1 Dermatology Division, Department of Medical Clinics, Ribeirão Preto Medical School, University of São Paulo, Ribeirão Preto, Brazil; 2 Department of Nefrology, VHP Saúde, Ribeirão Preto, São Paulo, Brazil

**Keywords:** Cocaine-related disorders, crack cocaine, drug toxicity, hand dermatoses

## Image in medicine

A 39-year-old male patient was admitted at the emergency room with headache, dry cough, and dyspnea three days ago. Dermatological examination revealed yellow and black-stained fingers in the first two fingertips of both hands.There were also a middle phalanx thermal burn with an ulcer on the third left finger. The patient did not have mental disorder. After the patient had been asked about the presence of the signs of thermal burn injuries, he reported having burned the digits by holding the pipe used to smoke crack. The diagnosis of fingertip thermal burn in crack cocaine user was made. As a powerful stimulant, crack use can elicit an extreme euphoria. As a smoked form of cocaine, crack cocaine use results in near immediate effects because the drug is readily absorbed from the lungs into the bloodstream. Binge use of crack can result in a psychotic, over-stimulated state accompanied by paranoia and compulsive behavior.S At the end of the binge, the person will normally crash, suffering agitation, depression and intense cravings. A person who has been smoking crack often has burned fingers or lips from holding onto the pipe while it heats up. Emergency health professionals should be trained to identify common dermatological signs of crack use because through them can be possible to suspect the diagnosis even if the patient omits his/her dependence. It must be emphasized that addiction is progressive and can result in disability or premature death.

**Figure 1:**
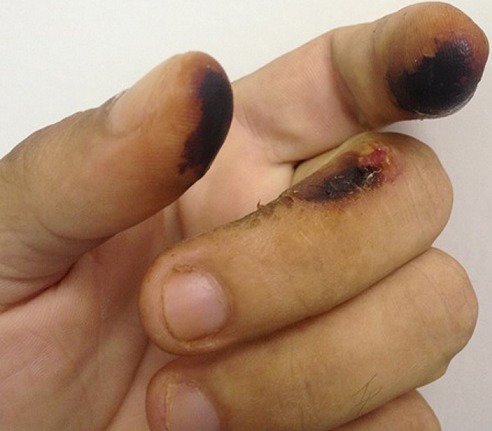
yellow and black-stained fingers with middle phalanx thermal burn and an ulcer on the third left finger

