# Corrigendum: Yellow and black-stained fingers in a patient with headache

**DOI:** 10.11604/pamj.2018.30.62.16070

**Published:** 2018-05-28

**Authors:** Fred Bernardes Filho

**Affiliations:** 1 Dermatology Division, Department of Medical Clinics, Ribeirão Preto Medical School, University of São Paulo, Ribeirão Preto, Brazil

**Keywords:** Corrigendum, cocaine-related disorders, crack cocaine, drug toxicity, hand dermatoses

## Abstract

This corrigendum corrects article “Yellow and black-stained fingers in a patient with headache” and its publication references ie The Pan African Medical Journal. 2017;28:318. doi:10.11604/pamj.2017.28.318.14333.

## Corrigendum

The original version of this article [1] had the author’s name wrongly abbreviated on pubmed ie Filho FB instead of Bernardes Filho F.
